# Supplementary material for: Hot spot prediction in protein-protein interactions by an ensemble system
Source: BMC Syst Biol. 2018 Dec 31;12(Suppl 9):132. doi: 10.1186/s12918-018-0665-8 (PMC6311905; doi:10.1186/s12918-018-0665-8)
Supplement: Supplementary file 2 — All relevant MATLAB code. A simple Matlab implement of our predictor. (ZIP 19,742 kb) [file 12918_2018_665_MOESM2_ESM.zip › Additional file 2/Read ASA/ASA website/BID/1NUNA.html]

 NetSurfP - Protein Surface Accessibility and Secondary Structure Predictions

  

|  |  |  |
| --- | --- | --- |
|  |  | NetSurfP - Protein Surface Accessibility and Secondary Structure Predictions  Technical University of Denmark |

  


---

```
# For publication of results, please cite:
# A generic method for assignment of reliability scores applied to solvent accessibility predictions.
# Bent Petersen, Thomas Nordahl Petersen, Pernille Andersen, Morten Nielsen and Claus Lundegaard
# BMC Structural Biology 2009, 9:51 doi:10.1186/1472-6807-9-51
#
# Column 1: Class assignment - B for buried or E for Exposed - Threshold: 25% exposure, but not based on RSA
# Column 2: Amino acid
# Column 3: Sequence name
# Column 4: Amino acid number
# Column 5: Relative Surface Accessibility - RSA
# Column 6: Absolute Surface Accessibility
# Column 7: Z-fit score for RSA prediction
# Column 8: Probability for Alpha-Helix
# Column 9: Probability for Beta-strand
# Column 10: Probability for Coil
E G  1NUNA                  1    0.886  69.728  -1.531   0.003   0.003   0.994
E R  1NUNA                  2    0.612 140.217  -0.887   0.018   0.047   0.935
E H  1NUNA                  3    0.461  83.801  -0.823   0.018   0.088   0.893
E V  1NUNA                  4    0.436  66.998  -0.771   0.018   0.088   0.893
E R  1NUNA                  5    0.438 100.348  -0.347   0.018   0.088   0.893
E S  1NUNA                  6    0.573  67.167  -0.387   0.056   0.142   0.802
B Y  1NUNA                  7    0.279  59.729  -0.463   0.052   0.084   0.864
E N  1NUNA                  8    0.642  94.047  -0.723   0.052   0.084   0.864
E H  1NUNA                  9    0.352  64.011  -0.146   0.056   0.142   0.802
B L  1NUNA                 10    0.146  26.788  -0.921   0.056   0.142   0.802
E Q  1NUNA                 11    0.548  97.873  -0.104   0.052   0.084   0.864
E G  1NUNA                 12    0.618  48.629  -1.720   0.052   0.084   0.864
E D  1NUNA                 13    0.345  49.772  -0.674   0.020   0.205   0.775
E V  1NUNA                 14    0.385  59.221  -0.430   0.021   0.451   0.528
B R  1NUNA                 15    0.232  53.105   0.538   0.021   0.756   0.223
E W  1NUNA                 16    0.255  61.400   1.503   0.011   0.918   0.071
B R  1NUNA                 17    0.077  17.633   0.926   0.006   0.962   0.032
B K  1NUNA                 18    0.102  21.043   0.846   0.006   0.962   0.032
B L  1NUNA                 19    0.017   3.113   1.474   0.011   0.918   0.071
B F  1NUNA                 20    0.063  12.604   0.520   0.011   0.918   0.071
B S  1NUNA                 21    0.024   2.801   0.372   0.021   0.756   0.223
B F  1NUNA                 22    0.185  37.170  -0.114   0.022   0.359   0.619
B T  1NUNA                 23    0.172  23.829  -0.455   0.020   0.205   0.775
E K  1NUNA                 24    0.328  67.470   0.211   0.004   0.197   0.799
B Y  1NUNA                 25    0.123  26.371   0.119   0.004   0.514   0.481
B F  1NUNA                 26    0.101  20.231   0.940   0.001   0.900   0.099
B L  1NUNA                 27    0.019   3.479   1.207   0.006   0.962   0.032
B K  1NUNA                 28    0.131  26.988   0.902   0.001   0.959   0.040
B I  1NUNA                 29    0.020   3.718   0.815   0.001   0.900   0.099
B E  1NUNA                 30    0.225  39.307   1.211   0.004   0.420   0.576
E K  1NUNA                 31    0.526 108.260  -0.270   0.004   0.085   0.910
E N  1NUNA                 32    0.717 104.969   0.209   0.005   0.015   0.979
B G  1NUNA                 33    0.142  11.207  -0.707   0.005   0.045   0.951
E K  1NUNA                 34    0.505 103.940   1.278   0.004   0.514   0.481
B V  1NUNA                 35    0.057   8.699  -0.419   0.003   0.718   0.279
E S  1NUNA                 36    0.468  54.885   1.141   0.022   0.552   0.426
B G  1NUNA                 37    0.078   6.115  -3.397   0.021   0.451   0.528
B T  1NUNA                 38    0.283  39.266  -0.436   0.022   0.552   0.426
E K  1NUNA                 39    0.441  90.673   0.074   0.005   0.336   0.660
E K  1NUNA                 40    0.485  99.682  -0.240   0.019   0.141   0.840
E E  1NUNA                 41    0.706 123.321  -0.521   0.018   0.047   0.935
E N  1NUNA                 42    0.576  84.268  -2.043   0.018   0.019   0.964
E C  1NUNA                 43    0.423  59.389  -0.983   0.005   0.045   0.951
E P  1NUNA                 44    0.471  66.849  -0.620   0.004   0.197   0.799
E Y  1NUNA                 45    0.364  77.894   0.242   0.004   0.514   0.481
B S  1NUNA                 46    0.049   5.755  -1.253   0.003   0.718   0.279
B I  1NUNA                 47    0.217  40.108   0.623   0.002   0.816   0.182
B L  1NUNA                 48    0.079  14.373   0.734   0.001   0.959   0.040
B E  1NUNA                 49    0.168  29.315   0.812   0.001   0.959   0.040
B I  1NUNA                 50    0.083  15.337   0.382   0.001   0.959   0.040
E T  1NUNA                 51    0.251  34.869   1.126   0.001   0.959   0.040
B S  1NUNA                 52    0.074   8.673  -1.964   0.001   0.900   0.099
B V  1NUNA                 53    0.236  36.335   0.140   0.004   0.420   0.576
E E  1NUNA                 54    0.546  95.404  -0.259   0.005   0.262   0.733
E I  1NUNA                 55    0.369  68.284  -0.087   0.004   0.085   0.910
E G  1NUNA                 56    0.288  22.689  -0.841   0.004   0.197   0.799
B V  1NUNA                 57    0.209  32.139   1.297   0.002   0.816   0.182
B V  1NUNA                 58    0.027   4.119   0.908   0.001   0.959   0.040
B A  1NUNA                 59    0.116  12.805   1.127   0.001   0.959   0.040
B V  1NUNA                 60    0.030   4.688   0.154   0.001   0.959   0.040
E K  1NUNA                 61    0.248  50.952   1.108   0.001   0.900   0.099
B A  1NUNA                 62    0.090   9.962  -0.367   0.003   0.718   0.279
B I  1NUNA                 63    0.158  29.267  -0.242   0.021   0.279   0.699
E N  1NUNA                 64    0.413  60.522  -0.146   0.021   0.279   0.699
E S  1NUNA                 65    0.313  36.719  -0.376   0.021   0.279   0.699
B N  1NUNA                 66    0.158  23.160  -0.189   0.004   0.197   0.799
B Y  1NUNA                 67    0.192  41.009   0.437   0.004   0.514   0.481
B Y  1NUNA                 68    0.049  10.407   0.720   0.011   0.918   0.071
B L  1NUNA                 69    0.023   4.230   1.144   0.006   0.962   0.032
B A  1NUNA                 70    0.014   1.565   0.880   0.006   0.962   0.032
B M  1NUNA                 71    0.032   6.383   0.121   0.001   0.900   0.099
B N  1NUNA                 72    0.119  17.348   0.293   0.004   0.420   0.576
E K  1NUNA                 73    0.598 123.029  -0.153   0.004   0.085   0.910
E K  1NUNA                 74    0.573 117.887   0.159   0.005   0.015   0.979
B G  1NUNA                 75    0.123   9.664  -1.487   0.004   0.138   0.858
E K  1NUNA                 76    0.266  54.798   0.770   0.003   0.718   0.279
B L  1NUNA                 77    0.041   7.489  -0.659   0.001   0.900   0.099
B Y  1NUNA                 78    0.145  30.901   1.086   0.001   0.900   0.099
B G  1NUNA                 79    0.049   3.848  -2.413   0.011   0.918   0.071
B S  1NUNA                 80    0.197  23.135   0.549   0.021   0.756   0.223
E K  1NUNA                 81    0.505 103.796  -0.360   0.023   0.655   0.322
E E  1NUNA                 82    0.406  70.911  -0.093   0.005   0.336   0.660
B F  1NUNA                 83    0.257  51.600  -0.350   0.004   0.138   0.858
E N  1NUNA                 84    0.325  47.521   0.194   0.005   0.045   0.951
E N  1NUNA                 85    0.597  87.372  -0.034   0.018   0.047   0.935
E D  1NUNA                 86    0.611  88.016   0.698   0.053   0.043   0.903
B C  1NUNA                 87    0.031   4.395  -0.166   0.069   0.386   0.545
E K  1NUNA                 88    0.301  61.916   1.717   0.021   0.756   0.223
B L  1NUNA                 89    0.083  15.234   0.291   0.018   0.846   0.136
E K  1NUNA                 90    0.320  65.865   1.939   0.072   0.855   0.073
B E  1NUNA                 91    0.116  20.248   0.112   0.072   0.855   0.073
E R  1NUNA                 92    0.378  86.654   1.119   0.085   0.775   0.140
B I  1NUNA                 93    0.171  31.635   0.652   0.169   0.612   0.219
E E  1NUNA                 94    0.363  63.434  -0.138   0.135   0.317   0.548
E E  1NUNA                 95    0.492  85.970  -0.891   0.118   0.150   0.732
E N  1NUNA                 96    0.367  53.758  -1.360   0.053   0.043   0.903
E G  1NUNA                 97    0.308  24.232  -0.243   0.053   0.043   0.903
B Y  1NUNA                 98    0.155  33.038   0.078   0.135   0.317   0.548
B N  1NUNA                 99    0.167  24.420  -0.301   0.087   0.683   0.230
B T  1NUNA                100    0.073  10.097   0.534   0.072   0.855   0.073
B Y  1NUNA                101    0.061  12.993   0.085   0.072   0.855   0.073
B A  1NUNA                102    0.143  15.715   0.691   0.085   0.775   0.140
B S  1NUNA                103    0.055   6.434  -0.765   0.085   0.775   0.140
B F  1NUNA                104    0.162  32.493   0.700   0.085   0.775   0.140
E N  1NUNA                105    0.275  40.216   0.531   0.087   0.683   0.230
B W  1NUNA                106    0.214  51.371   0.154   0.074   0.484   0.442
E Q  1NUNA                107    0.507  90.586  -0.579   0.066   0.296   0.638
E H  1NUNA                108    0.547  99.572  -0.909   0.056   0.142   0.802
E N  1NUNA                109    0.614  89.963  -1.535   0.018   0.047   0.935
E G  1NUNA                110    0.608  47.810  -1.232   0.018   0.019   0.964
E R  1NUNA                111    0.351  80.264   0.710   0.019   0.141   0.840
B Q  1NUNA                112    0.229  40.846   0.616   0.004   0.616   0.381
B M  1NUNA                113    0.043   8.624   1.122   0.018   0.846   0.136
B Y  1NUNA                114    0.056  11.903   0.618   0.011   0.918   0.071
B V  1NUNA                115    0.022   3.335   0.672   0.011   0.918   0.071
B A  1NUNA                116    0.029   3.240  -1.043   0.011   0.918   0.071
B L  1NUNA                117    0.029   5.328   0.320   0.003   0.718   0.279
E N  1NUNA                118    0.273  40.026   1.266   0.022   0.359   0.619
E G  1NUNA                119    0.489  38.492  -0.840   0.018   0.088   0.893
E K  1NUNA                120    0.670 137.881   0.654   0.018   0.047   0.935
E G  1NUNA                121    0.528  41.522  -1.208   0.005   0.045   0.951
E A  1NUNA                122    0.491  54.152   0.666   0.019   0.141   0.840
B P  1NUNA                123    0.219  31.090  -1.365   0.021   0.279   0.699
E R  1NUNA                124    0.394  90.112   0.047   0.021   0.279   0.699
E R  1NUNA                125    0.576 131.950  -0.396   0.020   0.205   0.775
E G  1NUNA                126    0.374  29.473  -1.362   0.018   0.088   0.893
E Q  1NUNA                127    0.497  88.764  -1.089   0.018   0.088   0.893
E K  1NUNA                128    0.417  85.715  -0.274   0.018   0.088   0.893
B T  1NUNA                129    0.224  31.041  -1.835   0.018   0.088   0.893
E R  1NUNA                130    0.415  95.012  -0.096   0.018   0.047   0.935
E R  1NUNA                131    0.627 143.560  -0.201   0.018   0.047   0.935
E K  1NUNA                132    0.628 129.221  -0.999   0.018   0.019   0.964
E N  1NUNA                133    0.236  34.550   0.168   0.018   0.047   0.935
E T  1NUNA                134    0.463  64.260   0.691   0.019   0.141   0.840
B S  1NUNA                135    0.135  15.869   0.263   0.022   0.359   0.619
B A  1NUNA                136    0.078   8.540  -0.595   0.021   0.451   0.528
B H  1NUNA                137    0.148  26.976   0.431   0.023   0.655   0.322
B F  1NUNA                138    0.123  24.646  -0.456   0.023   0.655   0.322
B L  1NUNA                139    0.122  22.283   0.926   0.023   0.655   0.322
B P  1NUNA                140    0.278  39.434   0.030   0.022   0.552   0.426
E M  1NUNA                141    0.252  50.525   1.096   0.022   0.552   0.426
E V  1NUNA                142    0.312  47.985  -0.801   0.021   0.451   0.528
B V  1NUNA                143    0.233  35.858  -0.705   0.021   0.279   0.699
E H  1NUNA                144    0.598 108.740   0.734   0.004   0.085   0.910
E S  1NUNA                145    0.792  92.787  -0.501   0.003   0.003   0.994

Explain the output.  Go back.
```

---
